# The effectiveness of colostral antibodies for preventing bovine leukemia virus (BLV) infection in vitro

**DOI:** 10.1186/s12917-018-1724-5

**Published:** 2018-12-29

**Authors:** Misako Konishi, Hiroshi Ishizaki, Ken-ichiro Kameyama, Kenji Murakami, Takehisa Yamamoto

**Affiliations:** 1 Epidemiology Unit, Division of Viral Disease and Epidemiology, National Institute of Animal Health, NARO 3-1-5 Kannondai, Tsukuba, Ibaraki 305-0856 Japan; 2 Grazing Animal Unit, Division of Grassland Farming, Institute of Livestock and Grassland Science, NARO. 768 Senbonmatsu, Nasushiobara, Tochigi 329-2793 Japan; 3 National Institute of Animal Health, NARO, Exotic Diseases Research Station Josuihoncho, Kodaira, Tokyo 187-0022 Japan; 4 Cooperative Department of Veterinary Medicine, Iwate University Faculty of Agriculture, 3-18-8 Ueda, Morioka, Iwate 020-8550 Japan

**Keywords:** Bovine leukemia virus, Colostral antibody, Proviral load, Prevention

## Abstract

**Background:**

Bovine leukemia virus (BLV) is the causative agent of enzootic bovine leukosis (EBL). The incidence of EBL in Japan is increasing annually; and the cases of EBL in cattle younger than 2 years old has been reported. Therefore, it is vital to find a method to control BLV infection, especially in young calves. In this study, to evaluate the protective ability of colostral antibodies against BLV infection, as well as the potential for BLV infection mediated by colostrum/milk, we investigated temporal fluctuations in the anti-BLV antibody titer and BLV proviral load (PVL) in colostrum/milk and peripheral blood of six infected dams during lactation. The association between PVL and antibody titer in colostrum and peripheral blood was then investigated using samples from a further twenty-seven cattle. Antibody concentrations were measured with a Syncytium-induction Inhibition Assay using colostral/milk whey and serum. PVL in peripheral blood and colostrum was measured by real-time PCR.

**Results:**

Colostral antibodies showed high inhibitory activity until day 3 of lactation. The antibody titer and PVL in peripheral blood showed lesser changes than those in colostrum/milk throughout lactation. The colostral antibody titer was significantly higher than the serum antibody titer in all samples, whereas the colostrum PVL was significantly lower than the blood PVL. The blood PVL showed a significant correlation with serum antibody titer, colostrum PVL, and colostral antibody titer. However, there were no major correlations between the serum and colostral antibody titers.

**Conclusions:**

This is the first report investigating the temporal changes in colostral antibody titer in terms of inhibiting BLV infection in vitro. The results of antibody detection by Syncytium-induction Inhibition Assay suggested that the protective activity of the colostral antibodies against BLV infection would be conferred by anti-BLV gp51 antibody. The high antibody titer of colostral whey suggests that colostral whey could be a potential source of antibodies with a low risk of infection in neonatal calves.

## Background

Bovine leukemia virus (BLV), a member of the family *Retroviridae*, is the causative agent of enzootic bovine leukosis (EBL). BLV infection is highly endemic in Japan [1]; despite of the low incidence of EBL (0.1–10% of BLV-infected cattle), the number of cattle diagnosed with EBL has increased in recent years. A nationwide investigation of the seroprevalence of BLV from 2009 to 2011 revealed that more than 20% of dairy cattle were already infected with BLV at less than 1 year old [1]. Although tumors caused by BLV are typically seen in animals over 3 years of age [2], infected calves have the potential to be affected by EBL at a younger age. Recently, several cases of EBL in cattle younger than 2 years old were reported in Japan [3]. The loss of young successor or fattening cattle can cause major economic damage to farms. Even if they are not affected by EBL, these animals would shed the virus within the farm for a longer time than cows infected at an older age. Therefore, preventing BLV infection in calves is vital for preventing the spread of the virus and reducing its economic impact. The target cells of BLV in cattle are B lymphocytes; thus, the blood, colostrum, and milk of infected cattle are known to be the major sources of infection [2, 4, 5]. On the other hand, several studies reported that anti-BLV antibodies found in the colostrum of BLV-infected cattle could protect newborn calves from BLV infection, both in vivo by experimentally infecting calves with BLV and through prospective studies without artificially infecting calves [6–9]. However, the mechanism of protection in the colostrum from infected dam is not clear, and its importance as an infection source has never been examined in vitro. Thus, the applicability of colostrum from infected dams to prevent BLV infection in neonatal calves remains controversial. In this study, to evaluate the protective ability of colostral antibodies against BLV infection, as well as the potential for BLV infection mediated by colostrum/milk, we investigated the fluctuations in the antibody titer and proviral load (PVL) in the colostrum/milk and peripheral blood of infected dams during the lactation period. Moreover, the association between PVL and the antibody titer in the colostrum and peripheral blood were investigated.

## Results

### Fluctuations in BLV antibody titer and PVL in colostrum/milk

**Fig. 1:**
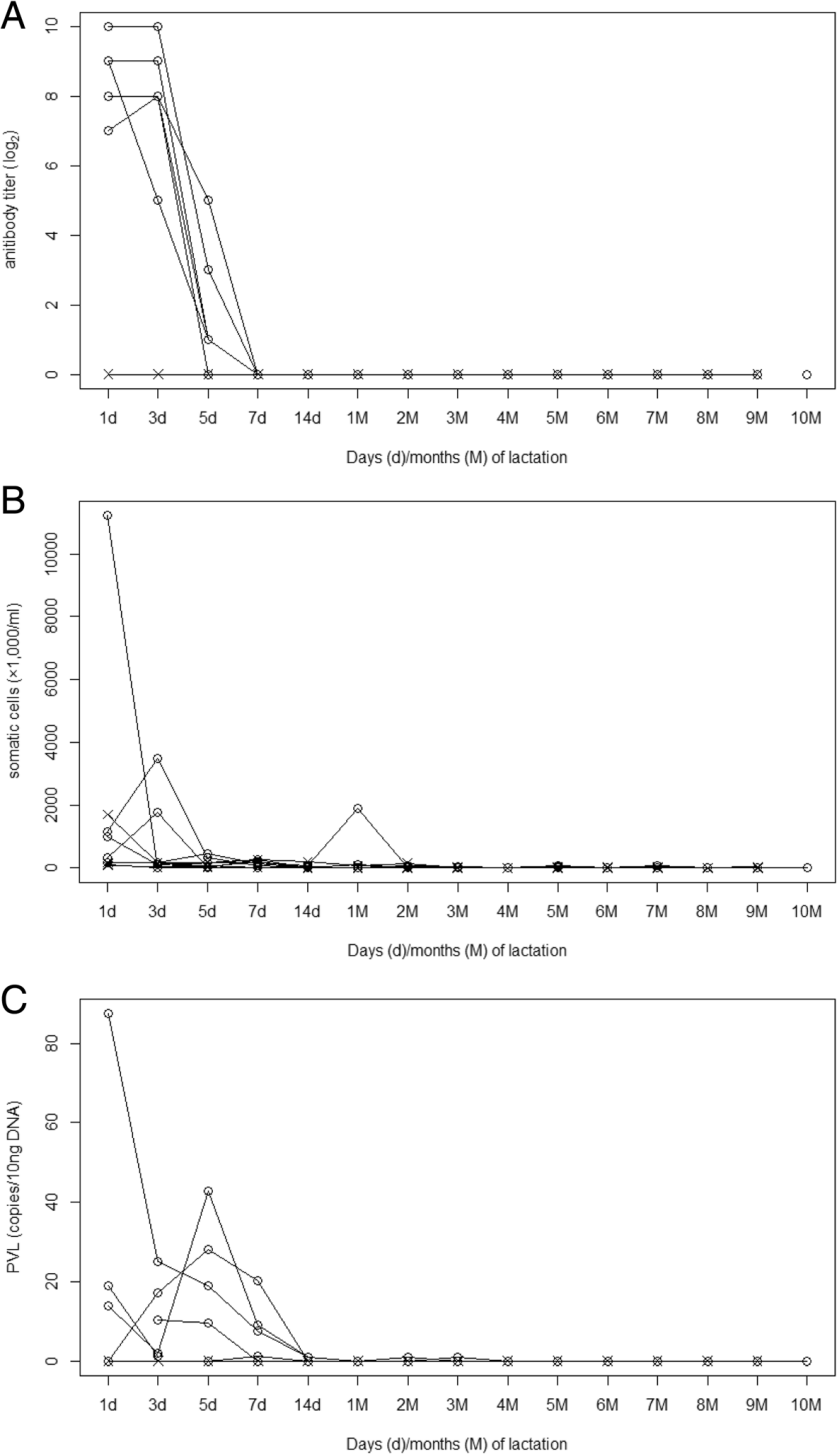
Temporal changes in anti-BLV antibody titer, somatic cell counts, and PVL in colostrum/milk. The temporal changes in (**a**) antibody titer; (**b**) somatic cell counts (SCC); and (**c**) PVL in colostrum/milk of BLV-infected cattle (○) and non-infected cattle (×)

The changes in antibody titer, somatic cells (SCs) count (SCC), and PVL in cattle colostrum/milk in experiment 1 are presented in Fig. 1. The antibody titer was highest at day 1 and 3 of lactation, decreased drastically from day 5, and became undetectable from day 7 until the end of sampling. SCC also achieved a peak at day 1 and 3 of lactation in three of six infected cattle and three of four non-infected cattle; levels were maintained at under 4.5 × 10^6^ cells/ml from day 5 in all cattle, except one infected cow, which showed levels of almost 2.0 × 10^6^ cells/ml at 1 month into the lactation period. Low levels of BLV proviral DNA were detected in infected cattle until day 7 of lactation, but concentrations were undetectable or 0.9–1 copy/10 ng DNA from day 14 until the end of the sampling period. The non-infected cattle all showed negative results for both Syncytium-induction Inhibition Assay (SIIA) and real-time PCR.

### Fluctuations in BLV antibody titer and PVL in blood

**Fig. 2:**
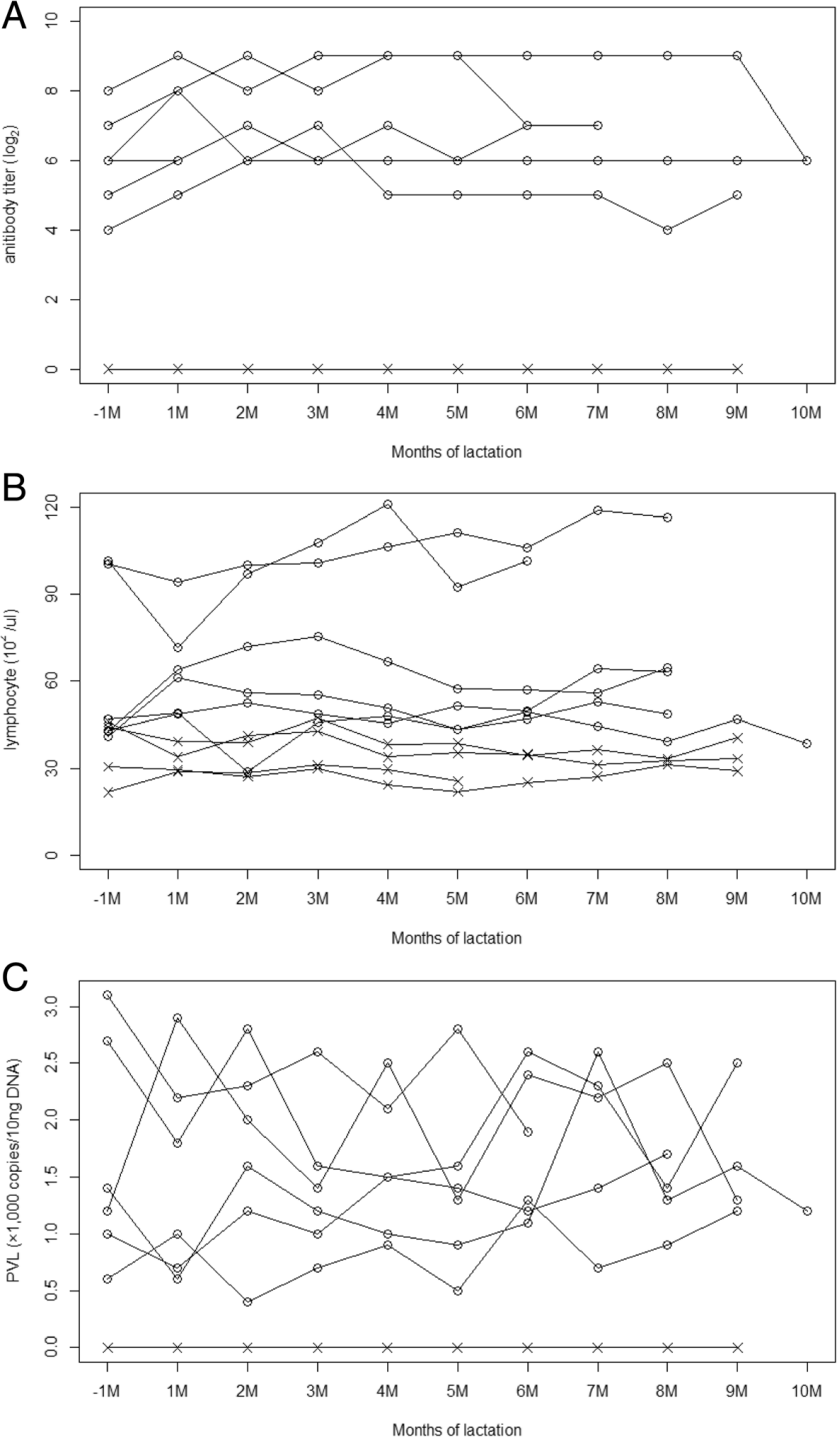
Fluctuations in anti-BLV antibody titer, lymphocyte counts, and peripheral blood PVL. Fluctuations in (**a**) antibody titer; (**b**) lymphocyte count; and (**c**) PVL in peripheral blood of BLV-infected cattle (○) and non-infected cattle (×). All measurements were taken from 1 month before the delivery date

The changes in the antibody titer, PVL, and number of lymphocytes in the blood of cattle in experiment 1 are presented in Fig. 2. The antibody titers in the sera of infected cattle ranged from 16 to 512 throughout the study period; no major decreases or increases were observed. The number of lymphocytes was approximately constant in each sample throughout the study period, ranging from 28.8–121.1 × 10^2^ /μl in infected cattle, and 21.7–47.7 × 10^2^ /μl in non-infected cattle. Fluctuations were observed in blood PVL, but levels were maintained at 0.7–3.1 × 10^3^ copies/10 ng DNA throughout the study period. The non-infected cattle all showed negative results for both SIIA and real-time PCR.

### Antibody titer and PVL in colostrum and blood samples

**Fig. 3:**
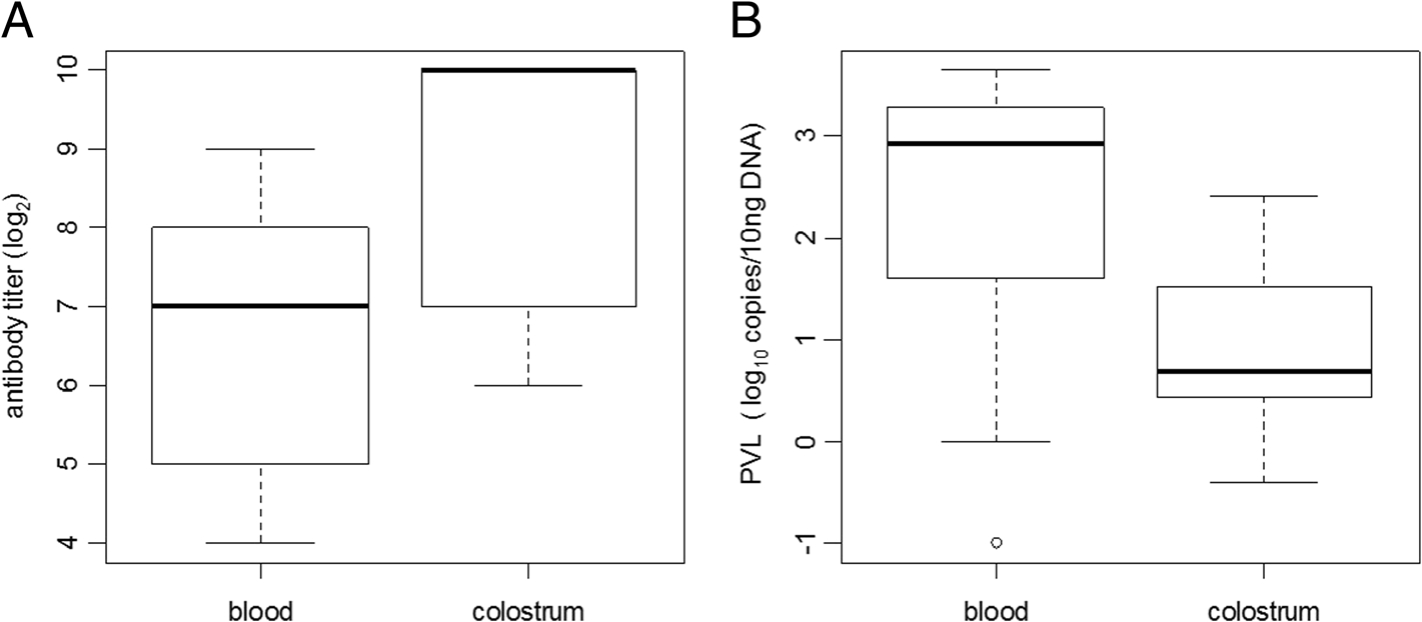
Comparison of anti-BLV antibody titer and PVL in colostrum and blood of infected cattle. The distribution of (**a**) anti-BLV antibody titers and (**b**) PVL between colostrum and peripheral blood

The colostral antibody titers from the twenty-seven infected cattle in experiment 2 ranged from 64 to 1024 (mean value: 644.7), whereas the serum antibody titers ranged from 16 to 512 (mean value: 134.5). The antibody titer in colostrum was significantly higher than that in blood (*p* < 0.05) (Fig. 3a). Proviral DNA was detected in 74.1% (20/27) of cattle in experiment 2. The PVL in colostrum ranged from 0 (not detected) to 254.6 copies/10 ng DNA (mean value: 21.6 copies/10 ng DNA), while that in blood ranged from 0.1 to 4363.3 copies/10 ng DNA (mean value: 1166.1 copies/10 ng DNA). The PVL in colostrum was significantly lower than that in blood (*p* < 0.05) (Fig. 3b).

### Correlation between PVL and antibody titer in blood and colostrum

**Fig. 4:**
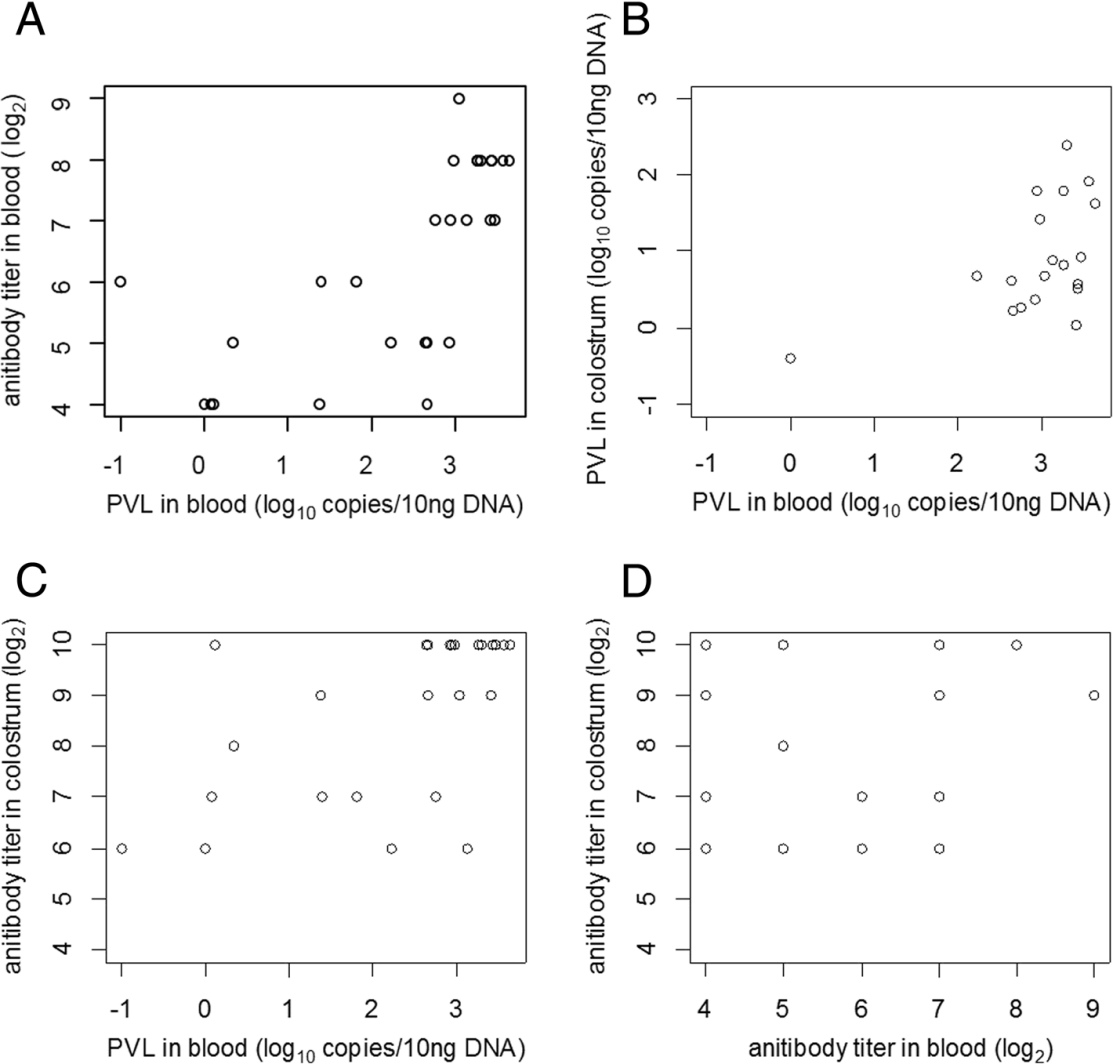
Relationship between PVL of BLV and antibody titer in blood and colostrum. Correlation between (**a**) PVL in bloo\\\\\d and serum antibody; (**b**) PVL in blood and PVL in colostrum; (**c**) PVL in blood and colostral antibody; and (**d**) serum antibody and colostral antibody

The correlations between PVL and antibody titer in blood and colostrum are presented in Fig. 4. Statistical analysis indicated that PVL in the blood showed a significant correlation with serum antibody titer, colostral antibody titer, and PVL in colostrum (*p* < 0.05). The correlation coefficients (r) between blood PVL and serum antibody titer, colostral antibody titer, and colostrum PVL were 0.69, 0.59, and 0.53, respectively. However, a significant correlation was not found between colostral antibody titer and serum antibody titer (*r* = 0.37, *p* = 0.06), and PVL in colostrum (*r* = 0.57, *p* = 0.06).

## Discussion

To evaluate the protective ability and infectivity of colostrum/milk from cattle infected with BLV, the temporal changes in antibody titer and PVL in colostrum/milk were investigated. Performing SIIA with whey samples allowed us to quantitatively measure the antibody titer in colostrum/milk. Although previous studies measured the antibody levels in colostrum and milk using ELISA [10, 11], we chose to use SIIA to measure antibody titers because one of the aims of this study was to determine the protective mechanism of colostral antibodies against BLV infection. Thus, this is the first report to investigate the temporal changes in the colostral antibody titer in terms of its protective ability against BLV infection in vitro. The results of antibody detection by SIIA revealed that colostrum from infected cattle inhibited syncytium induction caused by BLV infection. Syncytium formation results from cell-to-cell fusion caused by BLV gp51, an outer membrane glycoprotein [12, 13]. Previous studies showed the complement-dependent cytotoxicity of anti-gp51 antibody [14, 15], and previous studies hypothesized that the protective effect of BLV infection mediated by immunoglobulin (Ig) G would be conferred by anti-glycoprotein antibodies [16]. Thus, it can be concluded that the protective effect of colostral antibodies in BLV-infected cattle against BLV infection was conferred by anti-gp51 antibody, as has been observed in serum.

The results of experiment 1 revealed the chronological changes in PVL and antibody titer in both colostrum/milk and peripheral blood. The colostral antibody titer peaked at day 3 of lactation and rapidly decreased beginning on day 5. It is known that the total Ig levels in milk decline rapidly during the first 5 days after delivery [17, 18]. Thus, the decrease in anti-BLV antibody reflected the decrease in Igs during the transition from colostrum to mature milk. On the other hand, PVL in colostrum was lower than that in blood, and gradually decreased after day 5 of lactation. Most samples were negative for PVL after day 14 of lactation. These decreases in PVL in colostrum/milk seemed consistent with observed decreases in SCC. Since a portion of SCs were infected with BLV, a decrease in SCC would cause a decrease in PVL in colostrum/milk. Under normal circumstances, maternal antibodies and leukocytes contained in colostrum are absorbed through the intestinal epithelial cells of neonatal calves and delivered into their blood circulation. This capacity for absorption only lasts until 48 h after birth [19, 20]. Considering the low PVL in mature milk detected in this study and the decreased absorptive capacity of calf intestinal epithelial cells, it is possible that the role of mature milk from infected dams as a source of infection is not a major one. However, although the stage of lactation was unknown, a previous study detected BLV proviral DNA in the milk of infected cattle by quantitative real-time PCR [11]. Thus, the PVL in milk would vary with individual, and feeding milk with high PVL to calves without maternal antibodies could cause BLV infection to the calves. Since the infection threshold of PVL in milk remains unknown, the risk of BLV infection via ingesting raw milk should be evaluated in future studies.

The results of experiment 2 revealed that the antibody titer in colostrum was significantly higher than that in blood. Igs accumulate in the mammary gland during the prepartum dry period. Many different Igs, including IgG1, the predominant Ig in cattle, are selectively accumulated from blood circulation into the colostrum. It is known that this process causes 5- to 10-fold increases in the concentration of IgG1 in colostrum compared to maternal serum [17, 18]. Although the Ig class of the anti-gp51 antibody detected in this study was not investigated, we hypothesized that the increased antibody titer in colostrum compared to that in serum was the result of the selective accumulation of IgG1. On the other hand, PVL in colostrum was significantly lower than that in blood. In healthy cattle, the amount of PBL, SCC in colostrum, and SCC in mature milk of cattle are 4.9–12.0 × 10^3^ /μl, 1.0–3.0 × 10^6^ cells/ml, and 2.0 × 10^5^ cells/ml, respectively [19, 21, 22]. The predominant cell type in colostrum/milk is macrophages, whereas that in PBL is lymphocytes [19, 22]. Recent studies showed that the fraction of lymphocytes in bovine PBL is ~ 50% [23, 24]. On the other hand, the percentage of lymphocytes in colostrum SCs has been reported as 22–25%; in contrast, B lymphocytes, the target cells of BLV, account for less than 5% of the total lymphocytes [19, 21]. We therefore concluded that the low levels of PVL in colostrum were caused by the smaller proportion of B lymphocytes in colostrum than in blood.

A significant correlation between PVL in blood and serum antibody titer, PVL in colostrum, and colostral antibody titer were found in this study. Our findings are consistent with previous studies reporting that the anti-BLV antibody titer in serum should reflect PVL in blood [10, 25]. As described above, leucocytes and antibodies in colostrum were transferred from the blood. Thus, PVL in colostrum should be consistent with the level of PVL in blood. However, there were no significant correlations between antibody titer in blood and that in colostrum even though colostral antibodies were also accumulated from maternal blood. Some infected cattle had high colostral antibody titers despite their low serum antibody titers. The selective accumulation of Igs in colostrum would cause a major increase in the colostral antibody titer in these cattle and might be a cause of the unclear relationship between antibody titer in the blood and colostrum. In addition, we observed that 33.3% (9/27) of colostral samples completely inhibited syncytium induction at 1024-fold dilution, the highest dilution limit in this study; therefore, we may have underestimated the antibody titers in these samples. The selective accumulation of Igs in colostrum and the possibility of higher than expected antibody titers may also be a cause of the non-significant correlation between colostral antibody titer and PVL in colostrum.

In this study, a strong protective ability of colostral whey against BLV infection was found in vitro. This result suggests that colostral whey has potential use as a source of anti-BLV antibodies in neonatal calves. A notable advantage of colostral whey as source of anti-BLV antibodies is the low risk of BLV infection. Since milk whey does not contain SCs, cell-to-cell BLV infection would not occur. Moreover, cell-free virus particles in the whey could be inactivated by heat treatment. Kono et al. [16] parenterally administrated an IgG solution derived from the sera of sheep experimentally infected with BLV to non-infected sheep. They then inoculated the sheep with 50 μl whole blood from BLV-infected sheep several days after administration. Sheep with a passive antibody titer of 64 or more (determined by an agar gel immunodiffusion test) were not infected with BLV by the inoculation [16]. Based on those results, we hypothesized that colostral whey antibodies could become a source of passive antibodies in calves by feeding them with colostral whey immediately after birth. However, breastfeeding is known as one of the major routes of mother-to-child transmission of HTLV-1, the related virus of BLV, even though maternal antibodies are present in breast milk [26]. Therefore, the actual protective ability of colostral antibodies must be evaluated in vivo. Moreover, the relationship between the dosage of antibodies in colostrum and passive antibody titers in neonatal sera, the relationship between serum antibody titer and its subsequent protective effects, the duration of antibody effectiveness, and the absence of transmission by heat-inactivated colostrum whey must be investigated in vivo in order to verify the utility and safety of colostral whey as an antibody source in BLV-infected cattle. It is necessary to find donor cattle with high colostral antibody titers to prepare sufficient doses of colostral antibody; a high blood PVL would be a useful index for the selection of donor cattle before delivery.

## Conclusions

In this study, a strong protective activity of colostral antibodies against BLV infection was found in vitro. We considered that the protective effect of colostral antibodies in BLV-infected cattle against BLV infection was conferred by anti-gp51 antibody, as has been observed in serum. This result suggests that colostral whey has potential use as a source of anti-BLV antibodies in neonatal calves.

## Methods

### Animals and sample collection

In experiment 1, to investigate the temporal fluctuations in anti-BLV antibody titer in colostrum/milk and serum over the lactation period, colostrum/milk and blood samples were collected from six BLV-infected cattle and four non-infected cattle raised in a BLV-infected farm in Japan in 2014–2015. Blood samples were collected once a month from 1 month before the estimated date of delivery until the dry period; serum and peripheral blood leukocytes (PBL) were then extracted from the samples. Colostrum/milk samples were collected on day 1, 3, 5, 7, and 14 of lactation, and once a month from 1 month after the start of lactation until the dry-off date.

In experiment 2, twenty-seven BLV-infected cattle from a different BLV-infected farm in Japan were tested to examine the correlation between antibody titer and PVL. Colostrum was sampled at day 1 of lactation for each cow. In accordance with the owner’s intention, blood collection from all cattle in experiment 2 was conducted on the same day. The time between blood collection and colostrum collection ranged from 0 to 6 months (mean: 2.7 months).

All cattle in this study were tested for BLV infection using a commercial Enzyme-linked immunosorbent assay (ELISA) kit (JNC, Tokyo, Japan) and real-time PCR. Cattle that were positive for both tests were classified as infected, and cattle that were negative for both tests were classified as non-infected. Real-time PCR was performed as described below. Serum and milk whey separation, as well as the isolation of PBL and SCs, were performed within 2 days after sampling. Then, each sample was stored at − 20 °C for further experiments.

### Preparation of somatic cells and milk whey

Colostrum/milk samples (40 ml) were centrifuged at 3000 rpm for 20 min at 4 °C. The fat layer was removed using a micro-spatula, and milk whey was transferred to a new container. The precipitate was collected to isolate SCs and suspended in 1 ml phosphate-buffered saline (PBS). After the cells were counted using a TC20 automated cell counter (Bio-Rad Laboratories, Hercules, CA, USA), the cell suspension was centrifuged at 2000 rpm for 5 min at 4 °C. PBS was removed and cells were stored at − 20 °C until DNA extraction. Whey samples were prepared by centrifuging 12 ml milk whey at 30,000 rpm for 60 min at 4 °C. The whey samples were stored at − 20 °C until antibody titration, described below.

### Leukocyte count

The number of leukocytes and lymphocytes in peripheral blood were counted using a veterinary hematology analyzer (Celltac Alpha, MEK-6450; Nihon-Kohden, Tokyo, Japan).

### PBL isolation

PBL was isolated from 5 ml EDTA-treated blood according to the method previously described by Asfaw et al. [27].

### DNA extraction

DNA was extracted from SCs and PBL using a commercial DNA extraction kit (DNeasy Blood & Tissue Kit; Qiagen, Hilden, Germany) according to the manufacturer’s instructions.

### Quantification of BLV PVL

To detect and quantify BLV proviral DNA, real-time PCR was performed using a TaqMan probe. Primers and probes were designed to target the BLV *tax* gene according to the complete BLV proviral sequence obtained via GenBank (accession No. K02120). Primers and probe were as follows: sense primer (BLVCG-tax-8008F), 5’-CCATGTGACCGGTTACACGTAT-3′ (nucleotides [nt] 8008–8029); antisense primer (BLVCG-tax-8093R), 5’-ACCAATTCGGACCAGGTTAGC-3′ (nt 8073–8093); and TaqMan MGB probe (BLVCGtax8034T_F), 5’-FAM-CAGTCCTCAGGCCTT-MGB-3′ (nt 8034–8048). A synthetic 186-bp DNA fragment was used as a positive control. The fragment, identical to nt 7958–8143 (K02120), was synthesized using GeneArt Strings DNA (Thermo Fisher Scientific, Waltham, MA, USA). Serial 10-fold dilutions of the positive control, ranging from 10 to 10^6^ copies/5 μl, were used to draw a standard quantification curve. PCR was carried out using the QuantStudio 3 Real-time PCR System (Thermo Fisher Scientific) with the Premix EX-Taq (Perfect Real Time) kit (Takara Bio Inc., Shiga, Japan) according to the manufacturer’s instructions at a final reaction volume of 25 μl. Each reaction mixture contained 0.25 μM each primer 0.1 μM TaqMan MGB probe, and 5 μl DNA sample. The concentration of DNA in each sample was measured using a NanoDrop ND-1000 (Thermo Fisher Scientific), and the amount of DNA in each well was adjusted to less than 100 ng/well. Amplification was performed according to the following conditions: initial denaturation at 95 °C for 30 s, followed by forty-two cycles of denaturation at 95 °C for 5 s and annealing at 60 °C for 34 s. The measured value was divided by the amount of DNA in each well, and PVL was expressed as copies/10 ng DNA.

### Cell culture

CC81 cells, a feline cell line with high susceptibility to BLV, as well as fetal lamb kidney cells persistently infected with BLV (FLK-BLV), were used to measure the anti-BLV antibody titers as described below. CC81 cells were grown in Eagle’s minimal essential medium (MEM) with 5% fetal bovine serum (FBS) and 10% tryptose phosphate broth. FLK-BLV cells were grown in Eagle’s MEM with 5% FBS.

### SIIA

SIIA was performed based as previously described by Kono et al. [14]. A rabbit complement (Standard Rabbit complement; Cedarlane Laboratories, Burlington, Canada) that had no inhibitory effects on syncytium formation in CC81 cells by FLK-BLV was used at a 1:10 dilution. FLK-BLV cells were adjusted to a concentration of 2 × 10^5^ cells/ml, and CC81 cells were adjusted to a concentration of 1 × 10^6^ cells/ml. Sera and whey samples were heated for 30 min at 56 °C and 60 °C, respectively. For the dilution of samples and complement, as well as cell suspension, MEM supplemented with 2% FBS (2% growth medium [GM]) was used. Equal volumes (25 μl) of serial twofold dilutions (1:2–1:1024) of samples, FLK-BLV cell suspensions, and rabbit complement were mixed in 96-well plates and incubated at 37 °C for 1 h in a CO_2_-rich environment. Then, 25 μl CC81 cell suspension was added to each well and the plates were incubated for a further 2 days. Samples were tested in duplicate, and as a syncytium-positive control, 2% GM was added to eight wells instead of samples. As a syncytium-negative control, 2% GM was added to a further eight wells instead of the FLK-BLV cell suspension. The culture was stained with Giemsa and examined microscopically. Wells containing no syncytium were classified as anti-BLV antibody-positive. The antibody titer was expressed as the reciprocal of the highest serum dilution that showed a positive reaction. The antibody titer of samples that showed complete inhibition of syncytium induction in both wells of the 1024-fold dilution was designated as 1024.

### Statistical analysis

Associations of the anti-BLV antibody titer and PVL with colostrum and blood were evaluated using the Wilcoxon signed rank test. Correlations between PVL in blood and antibody titer in blood and colostrum, as well as PVL in colostrum, were evaluated by Pearson’s product-moment correlation coefficient. A value of *p* < 0.05 was considered statistically significant in all analyses. All statistical analyses were performed using R version 3.3.1 [28].

## Abbreviations

BLV: Bovine leukemia virus
EBL: Enzootic bovine leukosis
ELISA: Enzyme-linked immunosorbent assay
FBS: Fetal bovine serum
GM: Growth medium
Ig: Immunoglobulin
MEM: Minimal essential medium
PBL: Peripheral blood leukocytes
PBS: Phosphate-buffered saline
PVL: Proviral load
SCC: Somatic cell count
SCs: Somatic cells
SIIA: Syncytium-induction Inhibition Assay
